# LINC00452 promotes ovarian carcinogenesis through increasing ROCK1 by sponging miR-501-3p and suppressing ubiquitin-mediated degradation

**DOI:** 10.18632/aging.103758

**Published:** 2020-11-09

**Authors:** Juan Yang, Wei-Gang Wang, Ke-Qiang Zhang

**Affiliations:** 1 Department of Gynecologic Oncology Ward 5, Hunan Cancer Hospital and the Affiliated Cancer Hospital of Xiangya School of Medicine, Central South University, Changsha 410006, Hunan, People’s Republic of China

**Keywords:** ovarian cancer, long non-coding RNA, LINC00452, miR-501-3p, ROCK1

## Abstract

Ovarian cancer refers to all sorts of cancerous growth that starts from the ovary. Dysregulation of long non-coding RNAs (lncRNAs) is associated with ovarian cancer development and progression. Cellular expression and localization of LINC00452 in ovarian cancer cells were detected by qPCR and FISH. The roles of LINC00452 in ovarian carcinogenesis were characterized by MTT, transwell and colony-formation assays *in vitro* as well as xenograft mouse model. The underlying mechanism was explored by microarray, RIP, Co-IP and luciferase reporter assays. This study identified a novel lncRNA LINC00452 being elevated in both ovarian cancer cells and tumor tissues in patients. Such aberrant expression of LINC00452 was negatively correlated with relapse-free survival of ovarian cancer patients. Overexpression of LINC00452 potentiated CaOV3 cell viability, migration and invasion *in vitro* as well as xenograft tumor growth *in vivo*. Evidence from the current study suggests that the carcinogenicity of LINC00452 is partially due to competitive sponging of miR-501-3p followed with release of repression on the ROCK1, a key effector in Rho signaling pathway. Irrespective of its miRNA sponge function, LINC00452 is capable of preventing ROCK1 protein from ubiquitin/proteasome-mediated degradation via their mutual physical interaction. Our study makes LINC00452 a potential therapeutic target for ovarian cancer.

## INTRODUCTION

Ovarian cancer refers to a tumor with malignant cells being found inside, near or on the outer layer of ovary. It ranks first in all malignant gynecological cancer in terms of mortality, of which the five-year survival of ovarian cancer patients is merely 30~50% [1]. One of the major reasons for such poor mortality lies in the fact that the ovary is located deep in the pelvic cavity, and thus the symptoms are concealed. Eighty percent of the diseased women have already become advanced by the time of presentation [2]. In addition, a high recurrence rate after treatment and the acquired resistance to chemotherapy drugs are also the causes of low survival rates in patients with ovarian cancer.

Despite the poor prognosis for patients with advanced ovarian cancer, there remains a chance for a cure in early detection [3]. The prevailing approaches for diagnosis of ovarian tumors have included regular gynecological examination, B-ultrasound scan, CA125 and CA199 serum tumor makers tests, pelvic magnetic resonance and so forth. Among them, combined CA125 test and ultrasonography are recommended for symptom-triggered ovarian cancer diagnosis, although their specificities for early ovarian cancer detection are not high. Therefore, there is an urgent necessity for identifying clinical diagnostic biomarkers of ovarian cancer with higher sensitivity and accuracy.

Long non-coding RNA (lncRNA) is a class of longer than 200 nucleotides RNA transcripts, which plays important roles in regulating epigenetic modification, post-transcription, translation and protein stability [4]. Emerging evidence indicates that aberrant expression or dysfunction of lncRNA correlates closely to various cancer development [5, 6]. In ovarian tumors, there are a large number of dysregulated lncRNAs. These include but are not limited to lncRNAs such as HOX transcript antisense intergenic RNA (HOTAIR) [7], inactive X chromosome-specifically expressed Xist [8], maternally expressed gene 3 (MEG3) [9], imprinted maternally expressed H19 [10]. Functionally, dysregulated lncRNAs promote cell proliferation, migration and invasion, inhibit cell apoptosis, induce chemoresistance, increase cell movement, adhesion and metastasis as well as enhance glycolysis [11]. With the advance of high-throughput sequencing technologies, the number of lncRNA transcripts with an aberrant expression in ovarian cancer tissue is soaring and outpacing the rate of functional annotations. Deciphering the fundamental functions of individual lncRNAs in normal cells and their roles in tumor development and progression is therefore imperative.

By conducting an unbiased prognosis- and expression-based screening in different ovarian cancer database, we identified a novel lncRNA LINC00452 being commonly upregulated in various ovary cancer cells and tissues. It promoted ovarian cancer cell proliferation, migration and invasion *in vitro* as well as xenograft tumor growth *in vivo*. The underlying mechanisms included both indirect derepression of *ROCK1* mRNA through sponging miR-501-3p and direct binding and stabilization of ROCK1 protein. Our results shed light on the implication of the novel lncRNA LINC00452 as a therapeutic target of ovarian cancer.

## RESULTS

### LINC00452 is upregulated in ovarian tumor tissues and cancer cells and correlates with worse relapse-free survival in patients

**Figure 1:**
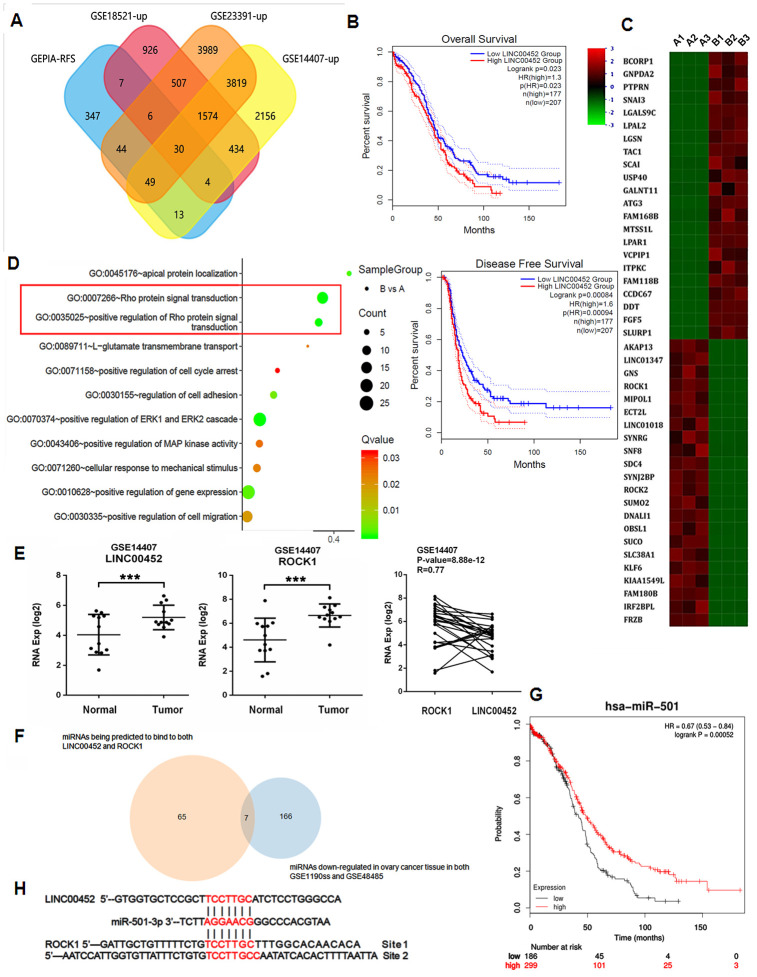
**Aberrant LINC00452 expression in ovarian tumor tissue and cancer cells.** (**A**) Venn diagram showing the overlapped genes being upregulated in ovarian tumor tissues and significantly associated with RFS. (**B**) Overall survival and RFS in ovarian cancer patients with higher LINC00452 level were shorter as demonstrated by Kaplan-Meier analysis. (**C**) Heatmap representation of the top differentially expressed genes (DEG) (2 < fold change < -2, p < 0.05) in LINC00452 knockdown (designated as group B) versus control (designated as group A) CaOV3 cells by microarray analysis. Color pattern represents row Z-score. (**D**) Bubble chart of enriched GOBP statistics. Rich factor is the ratio of the DEG number to the total number in a certain pathway. Q-value is a false discover rate corrected P-value ranging from 0 to 1. The color and size of the dots represent the range of the Q-value and the number of DEGs mapped to the indicated pathways, respectively. (**E**) LINC00452 and ROCK1 transcripts were elevated in ovarian tumor tissues compared with normal tissues, shown from microarray dataset GSE14407. Person’s correlation analysis showed a positive correlation in their expression in tumor. ***p < 0.001 versus normal tissues, student’s t-test. (**F**) Venn diagram showing the commonly downregulated miRNAs that are capable of binding to both LINC00452 and ROCK1 in ovarian tumors. (**G**) Survival curve of miR-501-3p in ovarian cancer patients. The overall survival in patients with lower miR-501-3p expression was shorter as indicated by Kaplan-Meier and log-rank tests. (**H**) Respective miR-501-3p binding sites in LINC00452 and ROCK1 as predicted in Targetscan database.

Many tumors are characterized as aberrant lncRNAs expression profile. To explore the roles of those essential yet unacknowledged lncRNAs in ovarian tumorigenesis, we performed an unbiased retrieval of all 500 genes associated with RFS from ovarian serous cystadenocarcinoma (OV) dataset in GEPIA2 [12]. These genes were then overlapped with the significantly upregulated genes in three independent human ovarian cancer microarray datasets GSE18521 [13], GSE14407 [14] and GSE23391 (https://www.ncbi.nlm.nih.gov/geo/query/acc.cgi?acc=GSE23391). As the Venn diagram in Figure 1A shown, a common set of 30 targets were found to meet the screening condition, among which 7 lncRNAs were identified (Supplementary Table 1). Interestingly, all other lncRNAs in the list were positively associated with RFS except for LINC00452, whose higher expression in ovarian surface epithelia correlates however, with worse RFS and overall survival in patients (Figure 1B). We then confirmed by qPCR that LINC00452 was also significantly upregulated in all general ovarian cancer cell lines including OVCAR3, SKOV3, CaOV3, A2780 and HO-8910 cells in comparison to the normal ovarian epithelial cells IOSE80 with the highest abundance detected in CaOV3 cells (Supplementary Figure 1). Therefore, we chose CaOV3 for the rest of functional studies.

LINC00452 is a novel lncRNA that has never been studied. In pursuit of the hints for its function, we knocked down LINC00452 in CaOV3 cells, and performed microarray analysis on the differentially expressed genes (DEG). Notably, Rho-associated coiled-coil containing protein kinase 1 (*ROCK1*) was identified as one of the top down-regulated genes in LINC00452-deficient cells (Figure 1C). Correspondingly, Rho protein signal transduction and positive regulation of Rho protein signal transduction terms were also enriched by gene ontology biological process (GOBP) analysis (Figure 1D).

*ROCK1* encodes a protein serine/threonine kinase that is activated upon binding to a GTP-bound form of Rho. The ROCK family genes are involved in many aspects of the fundamental cellular functions including contraction, adhesion, migration, proliferation and apoptosis [15]. Accumulating evidence supports that *ROCK1* acts as an oncogene and participates in tumor development and progression [16]. Therefore, our microarray analysis data reminded that the high LINC00452 expression-associated aggravation in RFS of ovarian cancer might be at least partially attributed to derepression of *ROCK1* gene. To test it, we firstly validated our results from a cell line-based study in human ovarian cancer microarray datasets GSE14407 [14]. As expected, both LINC00452 and *ROCK1* were significantly upregulated in primary ovarian tumor tissues, and Person’s correlation analysis showed a positive correlation in their expression in tumor (Figure 1E).

It is proposed by the canonical competing endogenous RNA (ceRNA) theory that lncRNAs act as microRNAs (miRNAs) ‘sponges’ or ‘decoys’ to sequester competitively bindings to their natural targets and thus releasing target genes from miRNAs’ silencing [17]. We then asked whether LINC00452 and *ROCK1* share any common interacting miRNAs. To address this question, we predicted through TargetScan [18] to have found 1125 *ROCK1*- and 183 LINC00452-binding miRNAs, respectively, of which 65 were shared in common (Figure 1F). We also obtained 166 commonly down-regulated miRNA candidates in ovarian tumors by comparing microRNA profiling array datasets GSE119055 [19] and GSE48485 [20] (Figure 1F). Further intersection analysis of the two subsets yielded 7 overlapped miRNAs (Figure 1F and Supplementary Table 2). In view of the negative correlation of LINC00452 with OV RFS (Figure 1B), its target miRNAs were expected an opposite association instead. Accordingly, by performing an online tool of Kaplan-Meier plotter analysis [21], we finally identified miR-501-3p a potential target of LINC00452 in ovarian tumors for its positive correlation to the survival of ovarian cancer patients (Figure 1G).

Taken all these analytical data together, we hypothesized that miR-501-3p is a common binding miRNA for both LINC00452 and *ROCK1* (Figure 1H), and constrains *ROCK1* expression in normal ovarian tissue. Induced LINC00452 competitively sponges cytosol miR-501-3p, and consequently derepresses *ROCK1* to promote ovarian tumorigenesis.

### LINC00452 potentiates ovarian cancer cells viability, invasion and colony formation

**Figure 2:**
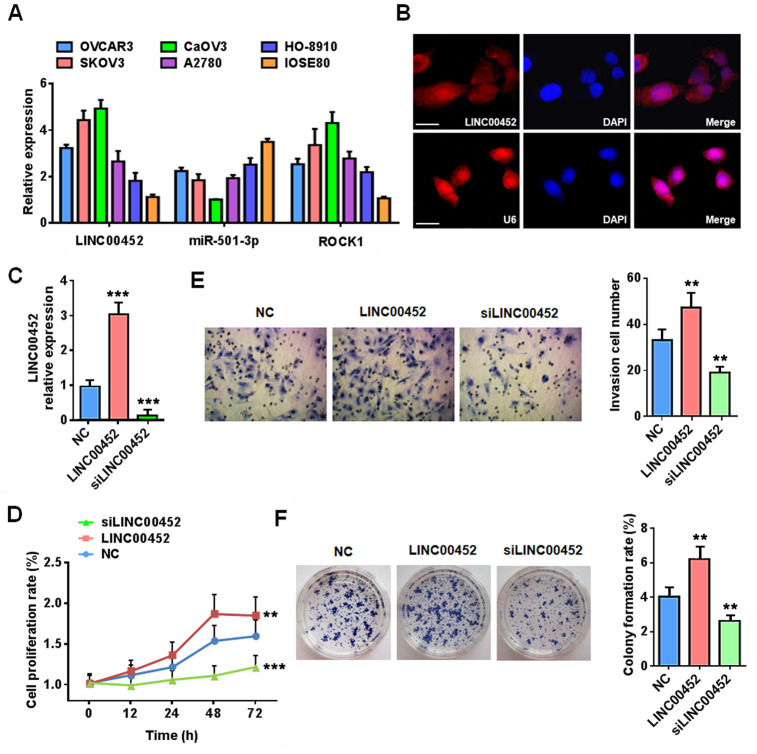
**LINC00452 promotes ovarian cancer cell carcinogenic properties.** (**A**) qPCR analysis displaying mRNA expression of LINC00452, miR-501-3p and ROCK1 in a panel of ovarian cancer cell lines and normal ovary epithelial cell line IOSE80. (**B**) RNA FISH results showing that LINC00452 was mainly located in the cytoplasm of CaOV3 cells. U6 was used as a control for nuclear localization. (**C**) Validation of LINC00452 expression in LINC00452-overexpressing and knockdown CaOV3 cells by qPCR. *** p < 0.001 versus control cells, one-way ANOVA test. (**D**) Time-dependent cell proliferation determined by MTT assay. LINC00452-overexpressing cells exhibited a growth advantage while LINC00452-knockdown cells displayed a growth defect over control CaOV3 cells. ** p < 0.01, *** p < 0.001 versus control cells, one-way ANOVA test. (**E**) Cell migration/invasion capacity determined by transwell assay. Upregulation of LINC00452 promoted whereas downregulation of LINC00452 compromised cell migration and invasion of CaOV3 cells. ** p < 0.01 versus control cells, one-way ANOVA test. (**F**) Colony-formation assay showing enhanced cell growth upon increasing LINC00452 and defective cell growth upon decreasing its expression. ** p < 0.01 versus control cells, one-way ANOVA test.

To validate our bioinformatics analytical data from ovarian cancer tissues, we determined the expression profiles of miR-501-3p and *ROCK1* in different ovarian cancer cells. In line with our assumptions, these cancer cells were also featured in general with lower miR-501-3p but higher *ROCK1* transcripts compared to normal IOSE80 cells (Figure 2A). Further FISH assay showed that LINC00452 is mainly located in the cytoplasm of CaOV3 cells (Figure 2B).

Next, we performed gain- and loss- of function assays to characterize the comprehensive effects of LINC00452 on CaOV3 cell propensities (Figure 2C). Strikingly, while a gain of LINC00452 enhanced cell proliferation, migration and invasion, loss of the gene exhibited the opposite effects as evidenced by results from MTT (Figure 2D), transwell (Figure 2E) as well as colony formation (Figure 2F) assays. Together, these data indicated that LINC00452 profoundly endows carcinogenic properties of ovarian cells.

### LINC00452 competitively sponges miR-501-3p to elevate *ROCK1* mRNA level

**Figure 3:**
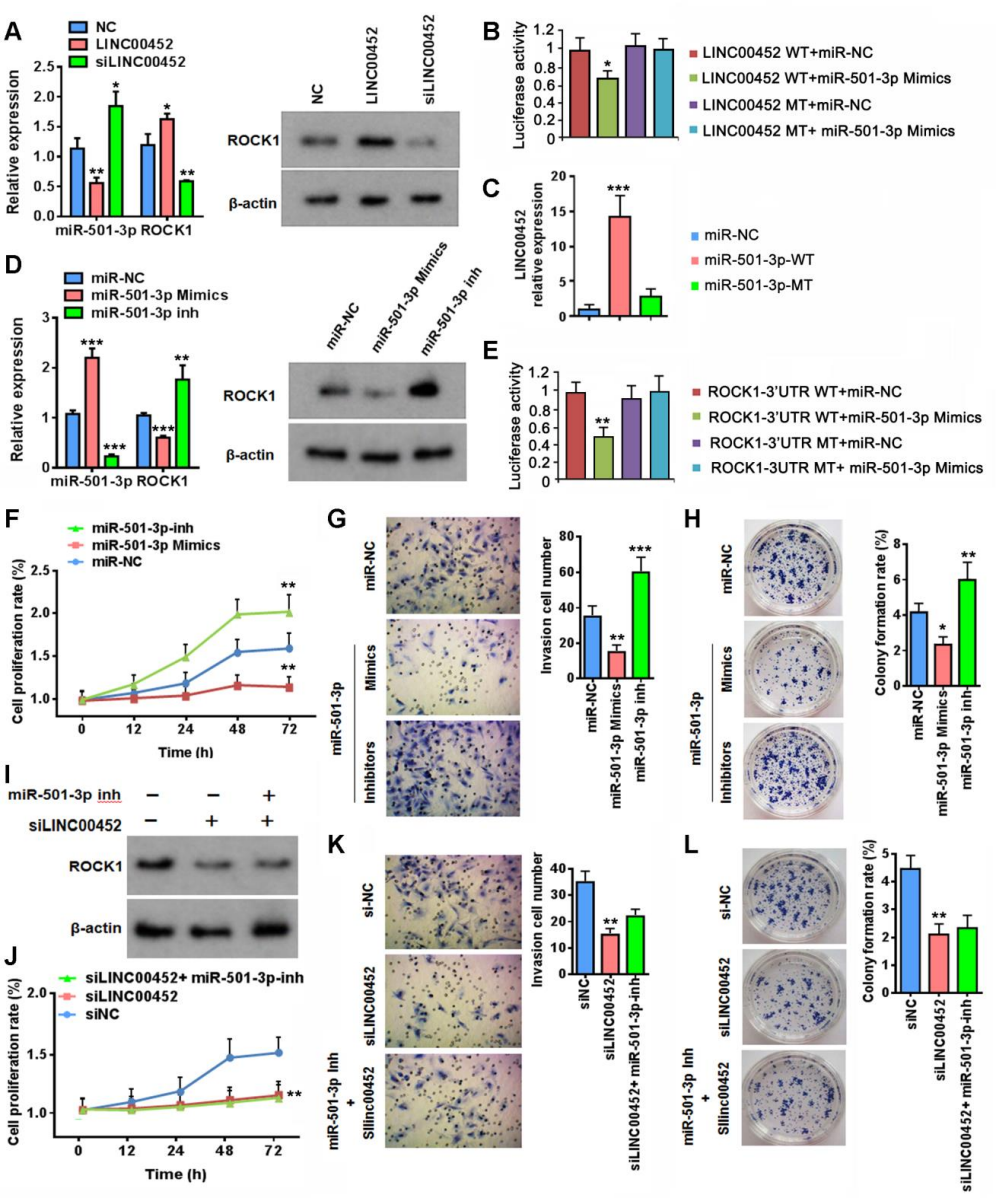
**LINC00452 regulates ROCK1 expression through competitive binding of miR-501-3p.** (**A**) Changes at RNA level of miR-501-3p and ROCK1 (left) as well as protein level of ROCK1 (right) upon overexpressing or knockdown LINC00452, respectively. * p < 0.05, ** p < 0.01 versus the corresponding vector-transfected control, one-way ANOVA test. (**B**) Luciferase reporter assay showing that miR-501-3p mimics reduced the luciferase activity of wild-type LINC00452 reporter, but not the construct with mutations at the binding sites. * p < 0.05 versus WT or MT vector-transfected controls, one-way ANOVA test. WT, wild-type; MT, mutant type. (**C**) RNA pull-down assay by incubating CaOV3 cell lysates with biotin-labeled wild-type (WT) or mutant (Mu) miR-501-3p followed with qPCR determining LINC00452 level from respective pull-down RNA extract. *** p < 0.001 versus miR-NC, one-way ANOVA test. (**D**) Determining ROCK1 RNA (left) and protein (right) expression upon transfection of miR-501-3p mimics or inhibitors (inh) in CaOV3 cells. ** p < 0.01, *** p < 0.001 versus control vector transfected cells, one-way ANOVA test. (**E**) Luciferase reporter assay showing that miR-501-3p mimics reduced the luciferase activity of wild-type ROCK1 reporter, but not the construct with mutations at the binding sites. ** p < 0.01 versus WT or MT vector-transfected controls, one-way ANOVA test. WT, wild-type; MT, mutant type. (**F**) Time-dependent cell proliferation determined by MTT assay. Cells transfected with miR-501-3p inhibitors exhibited advanced growth over the control vector-transfected CaOV3 cells. In contrast, miR-501-3p inhibitor-transfected cells displayed defective growth. ** p < 0.01 versus miR-NC, one-way ANOVA test. (**G**) Transwell assay determining cell migration/invasion capacity. Upregulation of miR-501-3p using mimics suppressed whereas downregulation of miR-501-3p by inhibitors promoted cell migration and invasion of CaOV3 cells. ** p < 0.01, *** p < 0.001 versus miR-NC, one-way ANOVA test. (**H**) Colony-formation assay showing defective cell growth upon transfecting miR-501-3p mimics and enhanced cell growth upon transfecting miR-501-3p inhibitors. * p < 0.05, ** p < 0.01 versus miR-NC, one-way ANOVA test. (**I**) Western blot assay determining ROCK1 protein changes upon downregulation of LINC00452 or combined decreases in both LINC00452 and miR-501-3p. (**J**–**L**) Inhibiting miR-501-3p failed in rescuing LINC00452 decrease-caused defects in CaOV3 cell proliferation (**J**), migration and invasion (**K**) as well as growth (**H**). ** p < 0.01 versus siNC group, one-way ANOVA test.

We proceeded to seek for experimental evidence supporting the proposed regulation of miR-501-3p and *ROCK1* by LINC00452 in CaOV3. As expected, LINC00452 overexpression reduced miR-503-3p transcription, whereas LINC00452 knockdown increased its transcription, respectively (Figure 3A). In contrast, overexpressing LINC00452 resulted in the induction of ROCK1 at both mRNA and protein levels, and vice versa (Figure 3A).

Next, we performed luciferase reporter assay to verify the predicted binding site between LINC00452 and miR-501-3p. It was clearly demonstrated that miR-501-3p mimics reduced the luciferase activity of wild-type LINC00452 reporter, but had no effect on the construct with mutations at the binding site (Figure 3B). The direct binding between LINC00452 and miR-501-3p was further testified by detecting highly enriched LINC00452 transcript in biotin-labeled wild-type but not the site-mutated miR-501-3p-pulldown cell lysates (Figure 3C). Similarly, we also demonstrated that transfection of miR-501-3p mimics significantly suppressed ROCK1 mRNA and protein expression. Instead, miR-501-3p inhibitors largely induced its transcription and translation as well (Figure 3D). Such regulation was mediated through the direct binding of miR-501-3p to the complementary sites in 3’-untranslated region (UTR) of *ROCK1* as evidenced by significantly decreased luciferase activity of wild-type *ROCK1*-3’UTR reporter in the presence of miR-501-3p mimics (Figure 3E). Targeted mutation in 3’-UTR of *ROCK1* fully abolished the inhibition of miR-501-3p mimics on reporter luciferase activity (Figure 3E). Functionally, reducing miR-501-3p by its inhibitor mimicked the effects of overexpressing LINC00452 by facilitating CaOV3 cell proliferation (Figure 3F), migration and invasion (Figure 3G) as well as colony-formation capability (Figure 3H). Instead, applying miR-501-3p mimics in CaOV3 cells resembled the inhibitory effects of LINC00452 on all these cancerous propensities (Figure 3F–3G).

So far, our results had suggested that LINC00452 acts as a miR-501-3p sponge controlling *ROCK1* expression and subsequently the outcome of RFS in ovarian cancer patients. Unexpectedly, concomitant addition of miR-501-3p inhibitor restored only mRNA (Supplementary Figure 2) but not protein expression of ROCK1 in LINC00452 knockdown CaOV3 cells (Figure 3I). Consistently, none of the compromised CaOV3 functions in proliferation, migration and invasion, as well as colony-formation upon knockdown of LINC00452 were rescued by inhibiting miR-50-3p (Figure 3J–3L). These data indicated that besides the transcriptional regulation of *ROCK1* by LINC00452 through competitively sponging miR-501-3p, the additional control at post-translational level was potentially involved.

### LINC00452 stabilizes ROCK1 protein by decreasing its ubiquitination

**Figure 4:**
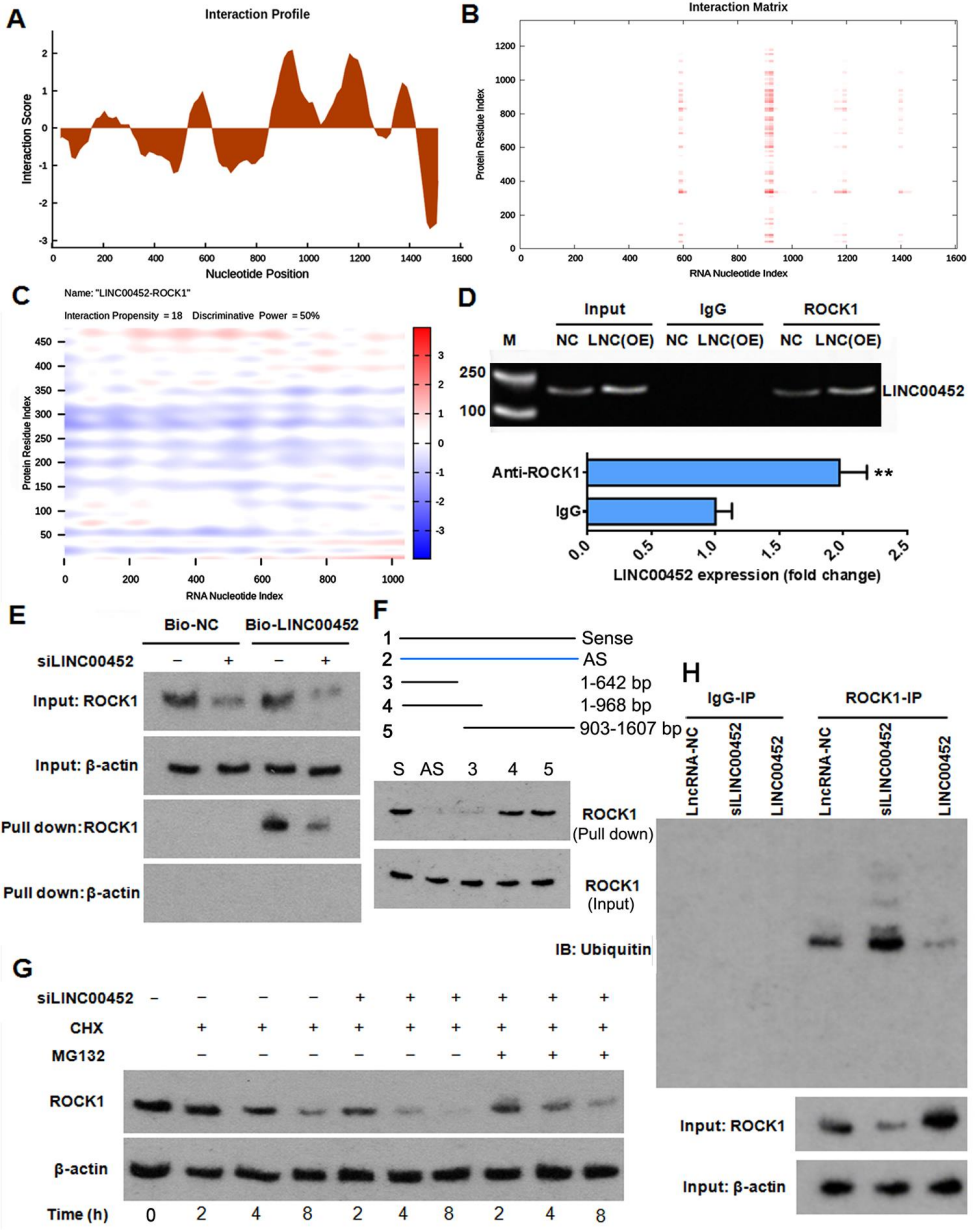
**LINC00452 protects ROCK1 from ubiquitin-proteasome-mediated degradation.** (**A** and **B**) interaction profile (**A**) and interaction matrix (**B**) showing catRAPID fragments-based prediction of interaction between LINC00452 and ROCK1. (**C**) catRAPID graphic output confirming the interaction of LINC00452 and ROCK1 as predicated by catRAPID fragments. (**D**) RIP results showing ROCK1 antibody specifically immunoprecipitated more LINC00452 from LINC00452-overexpressed CaOV3 cell lysates than control cells. Antibody against IgG was used as a negative control. M, DNA marker; LNC (OE), LINC00452 overexpression. ** p < 0.01 versus IgG group. (**E**) RNA pull-down assay using biotin-labeled LINC00452 yielded less ROCK1 protein when knocking down LINC00452 in CaOV3 cells. Beta-actin was used as a negative control indicating specificity of the interaction between LINC00452 and ROCK1. (**F**) In Truncated RNA pull-down assay, full-length LINC00452, antisense LINC00452, and truncated LINC00452 (1-190nt,1-1000nt,284-2036nt,190-2036nt) were synthesized. Results suggested that the binding region of ROCK1 protein in LINC00452 is most likely located in the 903-968 nt. (**G**) Western blot results showing knockdown of LINC00452 shortened the half-life of ROCK1, which could be reversed by blocking proteasome-mediated protein degradation with MG132. CHX, cycloheximide. (**H**) Co-immunoprecipitation of ROCK1 protein and ubiquitin. Knockdown of LINC00452 increased while overexpressing LINC00452 decreased ubiquitination of ROCK1 protein, respectively. Normal IgG was used as a negative control for IP.

To test whether there was LINC00452-related post-translational regulation of ROCK1 in ovarian cancer, we first conducted an *in-silico* prediction through catRAPID [22] the probability of any physical interaction between LINC00452 and ROCK1 protein. Interestingly, the analytical result of interaction profile file gave several highly scored domains in LINC00452 indicating a strong probability of interaction (Figure 4A). Information given in interaction matrix (Figure 4B) and interaction heat-map (Figure 4C) graphic output files further suggested that the protein domain of 325-380 amino acids in ROCK1 and the RNA regions ranging from 577 to 642 nucleotides (nt), 897 to 968 nt as well as 903 to 968 nt were most likely to mediate the mutual binding. This predicted protein-RNA interaction was subsequently validated by RNA immunoprecipitation (RIP) assay, which displayed a 2-fold increase of ROCK1-immunoprecipitated LINC00452 RNA in LINC00452-overexpressing CaOV3 compared to the control cells (Figure 4D). Similarly, RNA pull-down assay using biotin-labeled LINC00452 probe showed that less ROCK1 protein was pulled down when LINC00452 was knocked down in CaOV3 cells (Figure 4E). Results from truncated RNA pull-down assay further suggested that the binding region of ROCK1 protein in LINC00452 is most likely located in the 903-968 nt (Figure 4F). Together, these results provided strong evidence indicating the direct interaction between LINC00452 and ROCK1 protein.

Next, we asked whether the decreased ROCK1 protein in the absence of both LINC00452 and miR-501-3p (Figure 3I) was due to a sped-up protein degradation. We pre-treated LINC00452 knockdown CaOV3 cells with protein synthesis inhibitor cycloheximide (CHX), and determined the time-dependent ROCK1 protein degradation. Compared to the control group, knockdown of LINC00452 not only decreased the total amount of ROCK1 protein, which was related to suppression by miR-501-3p, but also dramatically shortened its half-life, indicating the decreased stability of ROCK1 in the absence of LINC00452 (Figure 4G). Interestingly, co-treatment with proteasome inhibitor MG132 delayed LINC00452 deficiency-caused ROCK1 degradation, suggesting that LINC00452 protects ROCK1 protein from a proteasome-dependent degradation (Figure 4G). As proteins being subjected to a degradation in proteasome are usually tagged with ubiquitin, we further tested the involvement of the ubiquitin-proteasome pathway in LINC00452-associated degradation of ROCK1 by Co-IP assay. Indeed, ubiquitinated ROCK1 in LINC00452 knockdown cells was largely increased, while overexpressing LINC00452 minimized ubiquitination of ROCK1 in CaOV3 cells (Figure 4H). As a whole, these data indicated that LINC00452 physically interacts with ROCK1 and protects it from ubiquitin/proteasome-mediated proteolysis.

### LINC00452 activates the downstream signals of ROCK1 protein

**Figure 5:**
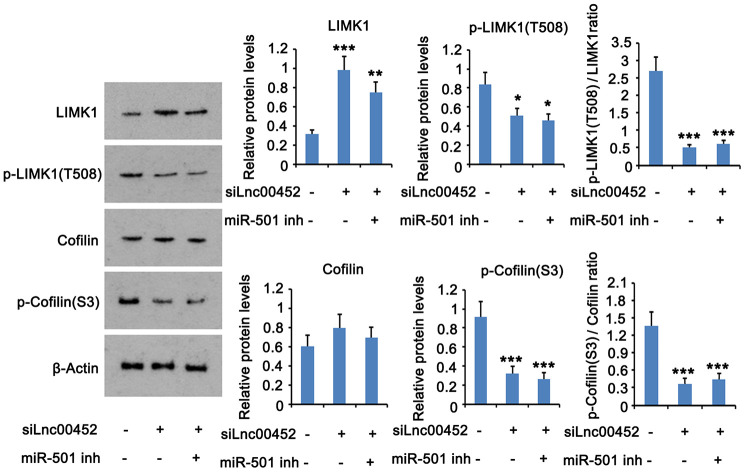
**LINC00452 activates the downstream signals of ROCK1 protein.** LINC00452 was knocked down alone or in combination with miR-501-3p knockdown. The protein levels of p-LIMK1, LIMK1, p-cofilin and cofilin were assessed in CaOV3 using western blot assay. * p < 0.05, ** p < 0.01; *** p < 0.001 versus control group.

Since both mRNA and protein levels of ROCK1 was increased by LINC00452, the downstream signals of ROCK1 protein was conjectured to be influenced by LINC00452 as well. As indicated by western blot assay, silencing LINC00452 increased LIMK1 protein level but decreased p- LIMK1 protein level, which cause the reduction of p-LIMK1/LIMK1 ratio (Figure 5). In addition, p-cofilin protein level and the ratio of p-cofilin/cofilin were reduced after LINC00452 knockdown. Suppression of the miR-501-3p partially restored the reduction of p-LIMK1/LIMK1 and p-cofilin/cofilin ratios.

### LINC00452 upregulation promoted xenograft tumor growth *in vivo*

**Figure 6:**
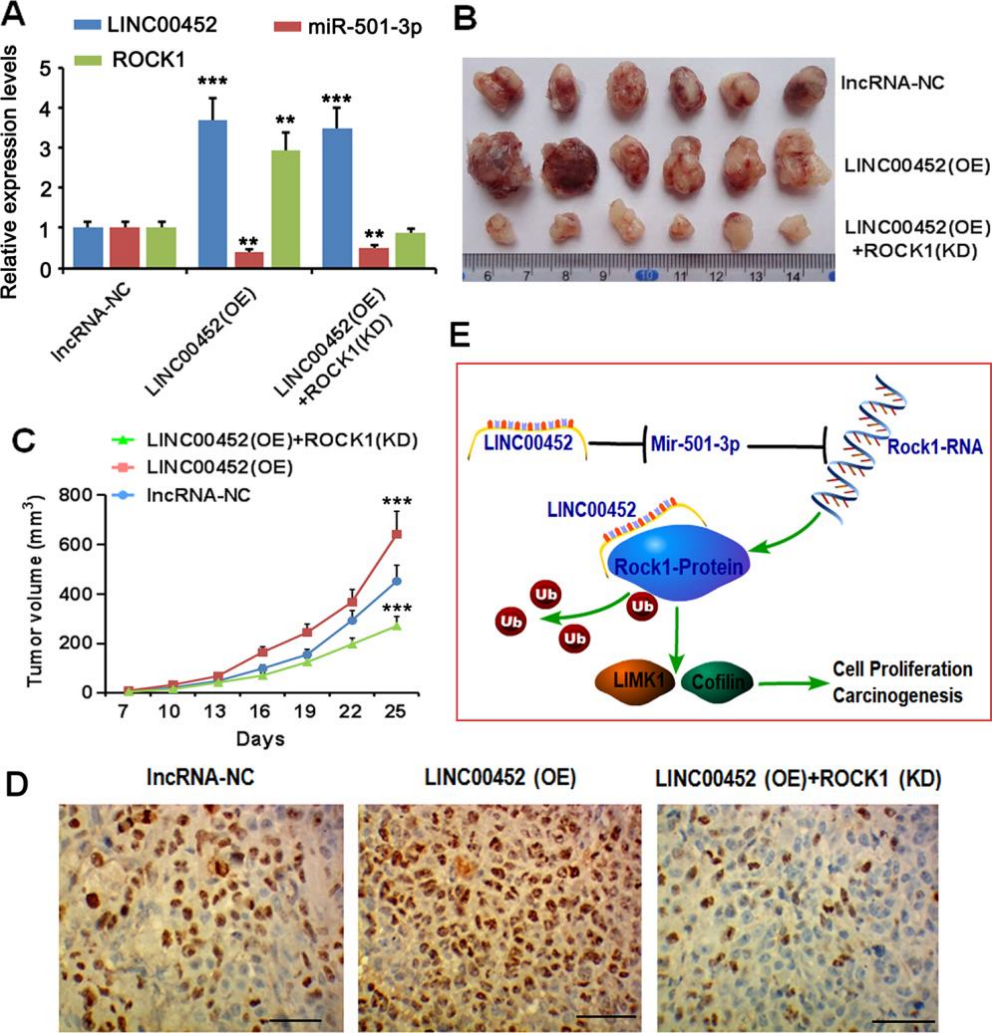
**LINC00452 promotes xenograft tumor growth in vivo.** (**A**) CaOV3 cancer cells with stable transfection of negative control lncRNA, LINC00452 as well as LINC00452 in combination with ROCK1-shRNA were subcutaneously injected into nude mice, respectively. PCR assay was performed to detect LINC00452, miR-501-3p and ROCK1 expression. (**B**) Tumor growth curves as recorded by measuring tumor volume. Overexpressing LINC00452 promoted tumor growth, which was abolished upon knockdown (KD) of ROCK1. *** p < 0.001 versus lncRNA-NC group. (**C**) Representative images showing xenograft tumors. (**D**) Immunohistochemistry results showing overexpressing LINC00452 promoted cell proliferation in tumors as indicated by more Ki67-positive cells. Knockdown (KD) of ROCK1 dramatically decreased number of Ki67-positive cells. (**E**) Diagram image showed that the carcinogenicity of LINC00452 is partially due to competitive sponging of miR-501-3p followed with release of repression on the ROCK1, a key effector in Rho signaling pathway. Irrespective of its miRNA sponge function, LINC00452 is capable of preventing ROCK1 protein from ubiquitin/proteasome-mediated degradation via their mutual physical interaction.

To explore the function of LINC00452 in ovarian tumorigenesis *in vivo*, CaOV3 cancer cells with stable transfection of negative control lncRNA, LINC00452 as well as LINC00452 in combination with ROCK1-shRNA were subcutaneously injected into nude mice, respectively. As expected, overexpressing LINC00452 significantly promoted xenografted tumor growth compared to the control group (Figure 6A–6C). However, simultaneous suppression of *ROCK1* dramatically slowed down the tumor growth rate despite of LINC00452 overexpression (Figure 6A, 6B). We further harvested tumors from each group for determining *in vivo* cell proliferation by immunohistochemical staining. In agreement with their tumor growth rates, LINC00452-overexpressing cell-derived tumors were overwhelmed with Ki67-positive proliferating cells (Figure 6D). Instead, cells in tumors grown from LINC00452-overexpressing but *ROCK1*-knockdown CaOV3 were much less proliferative as indicated by diminished Ki67 positive staining (Figure 6D). Taken together, the current study suggests that the carcinogenicity of LINC00452 is partially due to competitive sponging of miR-501-3p followed with release of repression on the ROCK1, a key effector in Rho signaling pathway. Irrespective of its miRNA sponge function, LINC00452 is capable of preventing ROCK1 protein from ubiquitin/proteasome-mediated degradation via their mutual physical interaction (Figure 6E).

## DISCUSSION

Gene regulatory networks are enormous and extensively distributed in cells, leading to intricate tumorigenesis mechanisms. A phenotypical difference in protein expression could be potentially rooted from multiple changes at the mRNA transcription, translation, and/or post-translation levels. In addition to mutations in protein-coding genes, emerging evidence indicates that aberrant expression of non-coding RNAs including lncRNAs and miRNAs accounts for many dysregulation in oncogenes and thus tumor aggressiveness and metastasis [23]. Our current study started from an unbiased screening of the upregulated human ovarian cancer signatures with strong correlations to RFS of OV to have identified the unique lncRNA candidate of LINC00452, whose expression is negatively associated with patients’ survival. To our knowledge, this was the first study disclosing the function of LINC00452 in cancer research. *In vitro*, LINC00452 was elevated in all general ovarian cancer cell lines including the serous ovary cancer cells lines OVCAR3, SKOV3 and CaOV3, as well as the ovary epithelial cancer cell lines A2780 and HO-8910 in comparison to the normal ovarian epithelial cell IOSE80. *In vivo*, it was also significantly upregulated in ovarian cancer tissues. Therefore, we consider the index of LINC00452 expression could be beneficial for the diagnosis and prognosis of ovarian cancer.

GO-BP analysis showed that Rho protein signal transduction pathway was dramatically affected upon LINC00452 knockdown in CaOV3 cells. The Rho subfamily members are of GTPase activity, and act as molecular switches controlling many essential cellular signal transductions. Rho proteins exert multiple biological effects by binding to their downstream target effector molecules, among which ROCK1 is one of the major effectors [24]. We speculated that alteration in Rho protein signal transduction pathway was resulted from the dramatic downregulation of its effector *ROCK1*. The Rho/ROCK signaling pathway affects profoundly cell migration through playing a central role in organizing actin cytoskeleton. ROCK regulates the activities of LIMK and cofilin (LIMK substrate), thus inducing cancer cell polarization, motility, and adhesion through promotion of actin–myosin filament bundling, myosin-driven contraction, actin-membrane linkage, and actin-filament stabilization. Indeed, the hyperactive ROCK1 signaling pathway was reported to promote migration and invasion of many types of cancer cells [25–27]. Therefore, the disordered Rho/ROCK1 signaling pathway can easily lead to tumor development and progression. Our study brings important insights into the function of LINC00452 through regulation on the key oncogene *ROCK1* in ovarian tumorigenesis.

It has been well-recognized that lncRNAs function as molecular decoys to sequester miRNA and indirectly regulate gene expression by inhibiting miRNAs from interacting with their target mRNAs [4, 28]. We provided solid bioinformatic and experimental data revealing the novel LINC00452/miR-501-3p axial controlling of *ROCK1* expression in ovary cancer cells. Of note, this is not the only regulator for *ROCK1* in ovarian tumor. For instance, it was reported that lncRNA NEAT1 promotes ovarian cancer cell metastasis through regulation of miR-382-3p/ROCK1 axis [29]. More recently, Pan et al. (2019) [30] demonstrated that LINC00339 facilitates ovary cancer cell proliferation, migration and invasion via miR-148a-3p/ROCK1 axis. All these studies indicate that eukaryotic cells develop a comprehensive regulatory network fine-tuning the activity of ROCK1, which is the central downstream effector for Rho protein signal transduction pathway. Although the beneficial effects including decreasing cell invasion, motility and invasion by direct ROCK inhibition have already been reported in a host of cancer cell-based studies [31–35], a general ROCK inhibition might however, simultaneously bring detrimental effects due to its involvement in a wide range of fundamental cellular functions including contraction, adhesion, proliferation and apoptosis [16]. Indeed, inhibiting ROCK by Y-27632 was shown to activate dormant MCF-7 breast cancer cells through disintegration of cell junctions coupled with the loss of E-cadherin and β-catenin in cell membrane [36]. In addition, ROCK inhibitor Y-27632-treated SW480 colon cancer cells exhibit enhanced migration due to changes in focal adhesions [37]. Targeting more specifically its upstream regulators instead of ROCK1 itself could be beneficial in avoidance of such detrimental side effects. Therefore, our study provides a potentially new and more specific target for ovarian cancer therapy.

Other than functioning as competitive endogenous RNAs, decoys or sponges, accumulating evidence indicates that lncRNAs can serve as master gene regulators at post-translational level as well [38]. We showed in the current study that LINC00452 knockdown decreased ROCK1 protein expression partially through increasing ubiquitin/proteasome-mediated degradation. This highlights the dual roles of LINC00452 in maintaining proper ROCK1 activity in normal cells. In view of the similar transcription mechanism of lncRNAs to mRNAs, future study identifying factors causing aberrant expression of LINC00452 is guaranteed for development of improved therapeutic strategy toward ovarian cancer.

In conclusion, we found that lncRNA LINC00452 was upregulated in ovarian cancer cells and tumor tissues, and strengthened the carcinogenic properties including cell proliferation, migration and invasion *in vitro* and xenograft tumor growth *in vivo*. This was accomplished through both miR-501-3p/ROCK1 axis as a ceRNA and alternatively, preventing ROCK1 from ubiquitin/proteasome-mediated degradation. Our study not only provides a novel marker for diagnosis and prognosis of ovarian cancer, but also offers a new target for ovary cancer therapy.

## MATERIALS AND METHODS

### Cell lines

Ovarian cancer cell lines including OVCAR3, SKOV3, CaOV3, A2780 and HO-8910 as well as ovarian epithelial cells IOSE80 were purchased from the Chinese Academy of Sciences Cell Bank (Shanghai, China). All cells were cultured in RPMI-1640 (Gibco, Grand Island, New York, USA) supplemented with 10% FBS (Sigma-Aldrich, St. Louis, MO, USA).

### Transfection and infection for overexpression or knockdown

5 x 10^4^ CaOV3 cells were seeded in 6-well plate on day before transfection. On the second day, LINC00452 plasmids (for LINC00452 overexpression), LINC00452-siRNAs (for LINC00452 knockdown), miR-501-3p-mimics (for miR-501-3p overexpression) miR-501-3p-inhibitors (for miR-501-3p inhibition) or negative control vectors (as control) were transfected using Lipofectamine^TM^ 2000 (Invitrogen; Thermo Fisher Scientific, Inc.) by following the instruction of manufacture. All sequences of the constructs were listed in Supplementary Table 3.

For *in vivo* xenograft experiment, lentiviral vectors expressing LINC00452, shROCK1 and the empty lentiviral vector were purchased from GeneChem (Shanghai, China). Virus infected CaOV3 cells were further selected by puromycin (2 μg/ml).

### RNA extraction and qPCR

To quantify LINC00452 and ROCK1 expression, total RNA from ovarian cancer cells and tissues were extracted using TRIzol reagent (Invitrogen; Thermo Fisher Scientific, Inc.), and reverse-transcribed using RevertAidTM H Minus First Strand cDNA Synthesis Kit (Fermentas). qPCR was then performed using SYBR Green PCR Master Mix (ABI 4309155) in ABI7900 Realtime-PCR machine. The primers used were β-actin (forward: 5’- CATGTACGTTGCTATCCAGGC-3’; reverse: 5’- CTCCTTAATGTCACGCACGAT-3’), LINC00452 (forward: 5’- CTCATGGCATGCAGAAGTGT-3’; reverse: 5’- GTCGAGCTTCACAGTGGACA-3’) and ROCK1 (forward: 5’-AACATGCTGCTGGATAAATCTGG-3’; reverse: 5’-TGTATCACATCGTACCATGCCT-3’). For analyzing miRNA expression, total RNA was purified using miRNeasy Mini Kit (Qiagen #217004) according to the instruction of manufacture. miRNA Q-PCR Detection Kit (GeneCopoeia #R0101L) was then applied for determining miR-501-3p expression. Primers used are has-miR-501-3p (forward: 5’- ACACTCCAGCTGGGAATGCACCCG-3’; reverse: 5’- CTCAACTGGTGTCGTGGAGTCGGCAATTCAGTTGAGCTTGCCCG-3’) and U6snRNA (forward: 5’- CTCGCTTCGGCAGCACA-3’; reverse: 5’- AACGCTTCACGAATTTGCGT-3’).

### Bioinformatics analysis

The 500 significant relapse-free survival (RFS)-associated genes were retrieved from GEPIA2-OV database (http://gepia2.cancer-pku.cn/#survival). Genes with logFC ≥ 0.5, p-value ≤ 0.05 in ovarian cancer database of GSE18521 and GSE14407 were considered being significantly upregulated and selected for intersecting with RFS-associated genes. *In silico* prediction of LINC00452-ROCK1 interaction was performed using online tools from catRAPID (http://s.tartaglialab.com/page/catrapid_group). The target miRNAs of LINC00452 and ROCK1 were predicted through computational algorithms in Targetscan database (http://www.targetscan.org/vert_72). The Kaplan Meier plotter (http://kmplot.com/analysis/index.php?p=background) was used to assess the effect of selected miRNAs on survival of ovarian cancer.

### Microarray analysis

The total RNA was extracted from scramble- or LINC00452-shRNA-transfected CaOV3 cells, respectively, and checked for integrity using Agilent 2100 Bioanalyzer. Only samples with RNA Integrity Number (RIN) of 8.0 and above were used for the transcriptome analysis by labeling and hybridization with Human Transcriptome Arrays 2.0 (Affymetrix, USA). Expression data were processed using Gene Expression Console software (Affymetrix, Santa Clara, USA). The significance of differentially expressed genes was determined using Transcriptome Analysis Console software (Affymetrix, Santa Clara, USA). Fold change ≥ 2 or ≤ -2 and a *p*-value ≤ 0.05 were defined as the threshold.

### Fluorescence *in situ* hybridization (FISH)

FISH was performed using oligonucleotide-modified probes for human lncRNA LINC00452 and a negative/scramble control. Briefly, CaOV3 cells were plated on autoclaved glass slides. On the second day, cells were fixed by 4% paraformaldehyde and permeabilized with 70% ethanol overnight at 4°C. Next day, permeabilized cells were washed with a solution of 10% formamide in 2x sodium citrate buffer (SSC), and proceeded with hybridization using LINC00452 or scramble control probes in a hybridization solution containing 10% formamide, 2x SSC and 10% dextran sulfate (w/v) overnight at 37°C in a humidified chamber. After a proper wash with 10% formamide in 2x SSC, cells were subjected to imaging using a confocal microscope.

### Cell viability assay

CaOV3 cells were seeded into 96-well plates (1 x 10^4^ cells/well), cell viability was measured at 12, 24, 48 and 72 h after seeding using MTT cell proliferation and Cytotoxicity Detection Kit (KeyGEN Biotech, #KGA312) by following the instruction of manufacture.

### Transwell assay

CaOV3 cells were seeded into the Matrigel (BD Biosciences, San Jose, CA)-coated upper chamber of 8.0 μm pore size Transwell apparatus (Corning, NY, USA) with serum-free medium. Growth medium supplemented with 10% FBS was added to the lower chamber as a chemoattractant. The cells were allowed to invade for 48 h at 37°C under 5% CO_2_. Cells invaded to the lower surface of filter were then fixed in 70% ethanol for 30 min followed with staining by 0.1% crystal violet for 10 min at 25°C.

### Colony-formation assay

CaOV3 cells were seeded in 60-mm dishes (200 cells/dish) in 3 ml complete growth medium. The medium was changed every other day for 2 ~ 3 weeks. Cells were then fixed with 75% ethanol for 30 min and subjected to 0.1% crystal violet staining and counting.

### Western blot

CaOV3 cells were lysed using RIPA buffer (1% NP-40, 0.1% SDS, 50 mM DTT) supplemented with protease inhibitor cocktail containing 2 μg/ml Aprotinin, 2 μg/ml Leupeptin and 1 mM PMSF. Cell lysates were subjected to ultrasonication followed with centrifuged for 10 min at 9,000 rpm to collect the supernatants. Equal amount of total proteins from each sample were resolved by 7% SDS-PAGE and electroblotted onto polyvinylidene difluoride membranes. Immunoblotting was performed with anti-ROCK1 (Abcam, #ab45171), anti-phosphorylated (p)-LIMK1 (Abcam, #ab194798), anti-LIMK1 (Abcam, #ab81046), anti-p-cofilin (Abcam, #ab12866), anti-cofilin (Abcam, # ab42824) and anti-β-actin (Santa Cruz, #sc-47778) antibodies.

### Luciferase reporter assay

Wild-type LINC00452 or ROCK1 3’UTR PCR fragments were cloned into psiCHECK-2 vector (Addgene, Inc., Cambridge, MA, USA) downstream of the firefly luciferase coding region within restriction sites XhoI and NotI (Takara Bio, Inc., Otsu, Japan). Corresponding mutations were introduced into the miR-501-3p binding site by site-directed mutagenesis using a fast mutation kit (New England BioLabs, Inc., Ipswich, MA, USA). The reporter plasmids were co-transfected with miR-NC, miR-501-3p mimics or miR-501-3p inhibitors, respectively, by LipofectamineTM 2000 (Invitrogen; Thermo Fisher Scientific, Inc.). Cells were lysed 24 h using Glo Lysis Buffer (E266A, Promega) after transfection, and the luciferase activity of each extract was assayed using Bright-Glo^TM^ Luciferase Assay System (E2620, Promega).

### RNA-immunoprecipitation (RIP) assay

RIP assay was performed in control and LINC00452-overexpressing CaOV3 cells using Magna RIP RNA-binding protein immunoprecipitation kit (Millipore, MA) according to manufacturer’s instructions. RNA extracted from total cell lysates, mouse IgG (as a negative control) and ROCK1 antibody (Abcam, #ab45171) immunoprecipitate were determined by qPCR using LINC00452 specific primers.

### RNA pull-down assay

CaOV3 cells were transfected with biotin-labeled miR-NC, wild-type has-miR-501-3p (5’-AAUGCACCCGGGCAAGGAUUCU -3’), mutated has-miR-501-3p (5’-AAUGCACCCGGCGUUAACUUCU -3’) or LINC00452, respectively. Forty-eight hours after transfection, cells were lysed on ice for 30 min in cell lysis buffer (10 mM KCl, 1.5 mM MgCl_2_, 10 mM Tris-HCl pH7.5, and 5 mM dithiothreitol) containing RNasin (Takara, Japan) and proteinase inhibitor cocktail (Roche, Sweden). Cell lysates were then incubated with streptavidin-conjugated beads overnight at 4^°^C. The beads were further cleaned using high salt washing buffer (5 mM Tris-HCl pH7.5, 0.5 mM EDTA, 1 M NaCl). For determining LINC00452, RNA was extracted from the remaining beads with TRIzol Reagent (Invitrogen; Thermo Fisher Scientific, Inc.) and analyzed by qPCR. For evaluating ROCK1 protein, total RNA was extracted with RIPA buffer and subjected to western blot analysis.

### Truncated RNA pull-down assay

According to the results of catRAPID prediction, the binding region of ROCK1 protein in LINC00452 is most likely located in the 903-968 nt and 577-642 nt. To identify the prediction, Full-length LINC00452, antisense LINC00452, and truncated LINC00452 (1-190nt,1-1000nt,284-2036nt,190-2036nt) were synthesized using the Biotin RNA Labeling Mix (Roche) by T7 RNA polymerase (Promega), treated with RNase-free DNase I (Promega) and purified with RNeasy Mini Kit (QIAGEN). The sequences were incubated with cell lysates at room temperature for 4 h, and then the biotin-labeled RNAs with their binding protein partner were pulled down by streptavidin magnetic beads (Thermo, USA) at 4 °C overnight. ROCK1 protein level in the binding proteins was determined using western blot assay.

### Co-immunoprecipitation (Co-IP)

Control, LINC00452- overexpressing and knockdown CaOV3 cells from 10-cm culture dishes were lysed with RIPA buffer, respectively. Human IgG (Bioss, #bs-0297P, 1:150) or ROCK1 antibody (Abcam, #ab45171, 1:50) were incubated separately with each cell lysate overnight at 4^°^C with gentle rotation. 20 μl Protein A/G agarose beads (Beyotime Biotechnology, #P1012) were added and incubated at 4^°^C with gentle rotation for 2 h. After the sequential wash with PBS and cell lysis buffer, the agarose beads were resuspended in 20 μl 1x SDS-PAGE loading buffer, and boiled for western blot analysis on Ubiquitin (Proteintech, 10201-2-AP, 1:200).

### Immunohistochemistry

Tumor tissues were fixed in 10% buffered formalin for 24 h and embedded in paraffin. The deparaffinized and rehydrated sections were blocked for endogenous peroxidase by 20 min incubation in 3% hydrogen peroxide followed with antigen retrieval at 121^°^C in citrate buffer (10 mM, pH6.0) for 10 min. After free cooling to room temperature, the sections were blocked for non-specific binding with normal goat serum (1:10) for 30 min at room temperature, and subjected to incubation with anti-Ki67 monoclonal antibody (1:100, Dako, Glostrup, Denmark) overnight at 4^°^C. The next day, the sides were washed and incubated with the biotinylated secondary antibody at 37^°^C for 30 min, and subsequently incubated with a 1:200 diluted streptavidin-biotin-peroxidase complex (Sigma, St. Louis) for 30 min. Reactive products were visualized with 3,3’-diaminobenzidene (DAB) as the chromogen, and nuclei were counter-stained with hematoxylin.

### *In vivo* xenograft experiments

The animal experiment was approved by the Ethical Committee for Animal Research of the Hunan Cancer Hospital and the Affiliated Cancer Hospital of Xiangya School of Medicine. Male BALB/c nude mice (4-6-week-old, n = 6 per group) were purchased from Beijing HFK Bioscience Co. Ltd (Beijing, China) and maintained under pathogen-free conditions. CaOV3 cells were stably transfected with control vector, LINC00452 or combined LINC00452 and ROCK1 shRNA, respectively, and then subcutaneously injected into BALB/c nude mice (1 x 10^6^ cells / injecting site). Tumor size was evaluated around 1 week after indicated time points.

### Statistical analysis

All data were expressed as the mean ± standard deviation of three independent experiments. Two-tailed Student’s *t*-test or one-way analysis of variance (one-way ANOVA) followed by Scheffe’s post-hoc test was performed using GraphPad Prism 6 software. Kaplan Meier survival analysis was used for analysis of survival rate and P-value was calculated by the log-rank test. *P* values less than 0.05 were considered significant.

## Supplementary Material

Supplementary Figures

Supplementary Tables
