# Supplementary material for: LINC00452 promotes ovarian carcinogenesis through increasing ROCK1 by sponging miR-501-3p and suppressing ubiquitin-mediated degradation
Source: Aging (Albany NY). 2020 Nov 9;12(21):21129–46. doi: 10.18632/aging.103758 (PMC7695380; doi:10.18632/aging.103758)
Supplement: Supplementary Figures [file aging-12-103758-s001..pdf]

SUPPLEMENTARY FIGURES

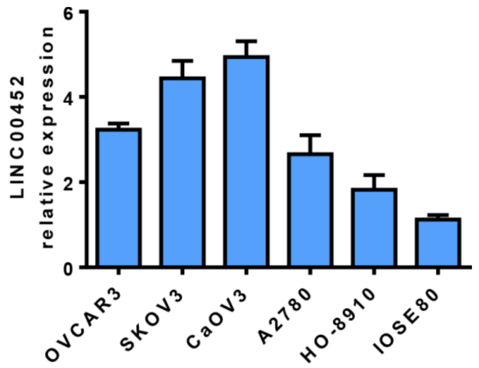

Supplementary Figure 1. The expression of LINC00452 in ovarian cancer cell lines and the normal ovarian epithelial cells.

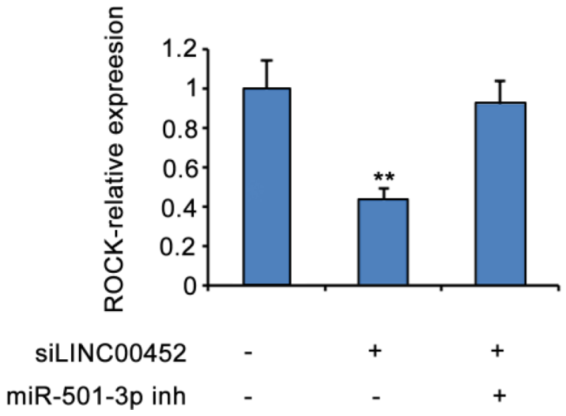

Supplementary Figure 2. Rock mRNA expression levels after silencing LINC00452 alone or together with miR-501-3p inhibition.
