# Supplementary material for: LINC00452 promotes ovarian carcinogenesis through increasing ROCK1 by sponging miR-501-3p and suppressing ubiquitin-mediated degradation
Source: Aging (Albany NY). 2020 Nov 9;12(21):21129–46. doi: 10.18632/aging.103758 (PMC7695380; doi:10.18632/aging.103758)
Supplement: Supplementary Tables [file aging-12-103758-s002..pdf]

SUPPLEMENTARY TABLES

Supplementary Table 1. Seven identified lncRNAs that are overlapped in three human ovarian cancer microarray datasets GSE18521, GSE14407 and GSE23391.

ID	RNA type	Size (bp)	Association with prognosis
LINC00452	LncRNA	1607	Negative
LINC01144	LncRNA	1740	Positive
DIAPH2-AS1	LncRNA		Positive
FOXP4-AS1	LncRNA		Positive
MIAT/ LINC00066	LncRNA		Positive
RP11-108L7.15	LncRNA		Positive
SLC25A5-AS1	LncRNA		Positive

Supplementary Table 2. Seven miRNAs that are overlapped in miRNA profiling array datasets GSE119055 and GSE48485.

	ID	adj.P.Val	P.Value	t	B	logFC	miRNA_ID
GSE119055	20504340	0.974826	8.69E-01	-0.16999	-6.42903	-0.02683	hsa-miR-600
GSE48485	610	0.612	0.091979	-2.63972	-4.044	-3.2123	hsa-miR-600
GSE119055	20504410	0.6163	1.28E-01	-1.68	-5.12077	-0.25892	hsa-miR-548d-3p
GSE48485	3927	0.97	0.883585	-0.16154	-5.022	-0.19657	hsa-miR-548d-3p
GSE119055	20506712	0.699492	2.00E-01	-1.3863	-5.50315	-0.85628	hsa-miR-1180-3p
GSE48485	1071	0.749	0.329214	-1.05393	-4.914	-1.89672	hsa-miR-1180
GSE119055	20503103	0.618973	1.31E-01	-1.66853	-5.13644	-2.21175	hsa-miR-485-5p
GSE48485	4131	0.604	0.087401	-2.17099	-3.988	-2.59367	hsa-miR-485-5p
GSE119055	20503878	0.013204	2.89E-05	-7.92849	2.979649	-2.6724	hsa-miR-501-3p
GSE48485	7976	0.798	0.434801	-0.81186	-5.33	-0.85363	hsa-miR-501-3p
GSE119055	20503872	0.652933	1.57E-01	-1.5494	-5.29586	-0.23384	hsa-miR-516a-3p
GSE48485	9475	0.574	0.039517	-3.91237	-3.802	-4.76101	hsa-miR-516a-3p
GSE119055	20504316	0.904389	6.24E-01	-0.50802	-6.3065	-0.26299	hsa-miR-548a-3p
GSE48485	11244	0.574	0.038362	-3.96387	-3.795	-4.82368	hsa-miR-548a-3p

Supplementary Table 3. Sequences of the constructs for LINC00452 knockdown, miR-501-3P overexpression and knockdown

Sequence name	Sense (5'-3')	Antisense (5'-3')
LINC00452-476	AGAAGAAGAGGCUGAAUCCTT	GGAUUCAGCCUCUUCUUCUTT
LINC00452-546	AUUUCCUAGGGAAACUCUGTT	CAGAGUUUCCCUAGGAAAUTT
LINC00452-1569	UUAUUCCACAGUUCAAGUGTT	CACUUGAACUGUGGAAUAATT
Negative control FAM	UUCUCCGAACGUGUCACGUTT	ACGUGACACGUUCGGAGAATT
miR-501 -3P-Mimics	AAUGCACCCGGGCAAGGAUUCU	
miR-501 -3P-inhibitors	AGAAUCCUUGCCCGGGUGCAUU	
Negative control RNAi	UUCUCCGAACGUGUCACGUTT	ACGUGACACGUUCGGAGAATT
